# Supplementary material for: Rab3A/Rab27A System Silencing Ameliorates High Glucose-Induced Injury in Podocytes
Source: Biology (Basel). 2023 May 9;12(5):690. doi: 10.3390/biology12050690 (PMC10215186; doi:10.3390/biology12050690)
Supplement: Supplementary file 1 [file biology-12-00690-s001.zip › SUPPLEMENTARY MATERIAL.pdf]

SUPPLEMENTARY MATERIAL

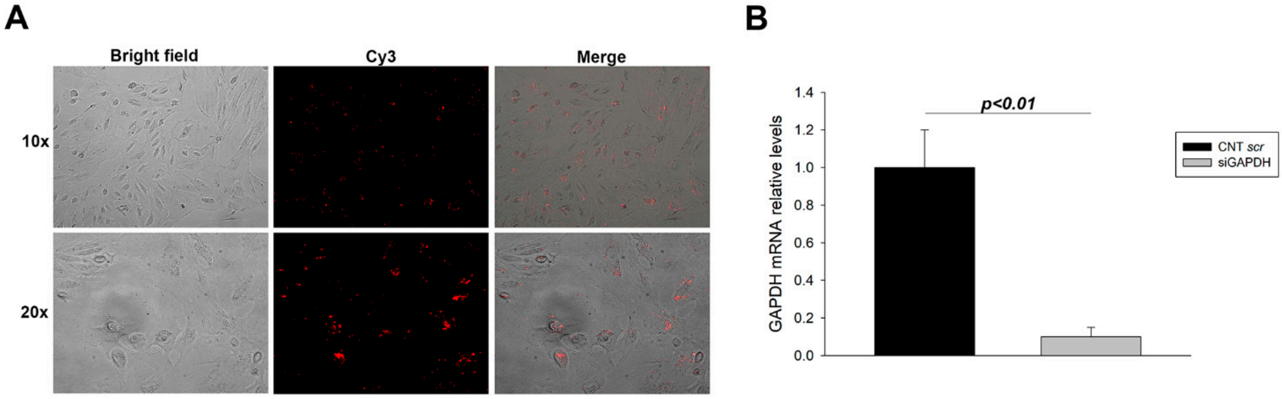

Supplementary Figure S1. Transfection efficiency of siRNAs in podocytes. (A) Microscopy images of bright field and fluorescence of Cy3 Dye-Labeled Negative Control at different magnifications (10x and 20x)**. (B)** GAPDH mRNA levels when using the GAPDH siRNA. CNT scr, control *scramble*; siGAPDH, siRNA of GAPDH. Data are shown as media±SEM. N=5 for each group**.** mRNA levels for CNT scr group are normalized to 1.

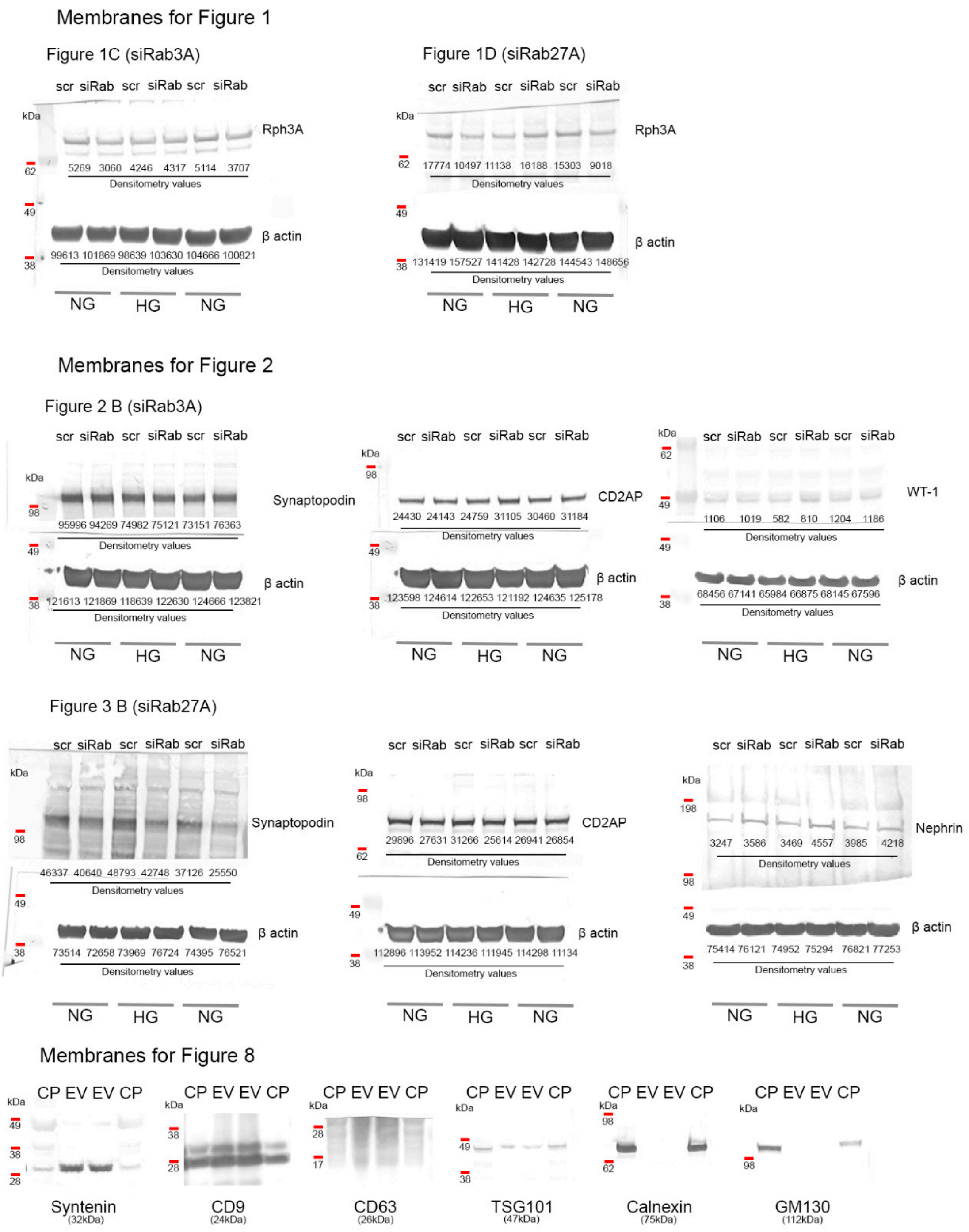

Supplementary Figure S2. Membrane blots from whole gel of the Figures 1,2,3 and 8. Membrane blots with molecular weight markers in red and densitometry readings of each band form all the figures with western blot experiments. Membranes from Figure 8 were no quantified, only for absence or presence of the protein, so do not have densitometry readings. scr: scramble; NG: normal glucose; HG: high glucose; siRab3A: Rab3A siRNA, siRab27A: Rab27A siRNA, siRab: Rab siRNA.
