# Supplementary figures and images for: Rab3A/Rab27A System Silencing Ameliorates High Glucose-Induced Injury in Podocytes
Source: Biology (Basel). 2023 May 9;12(5):690. doi: 10.3390/biology12050690 (PMC10215186; doi:10.3390/biology12050690)

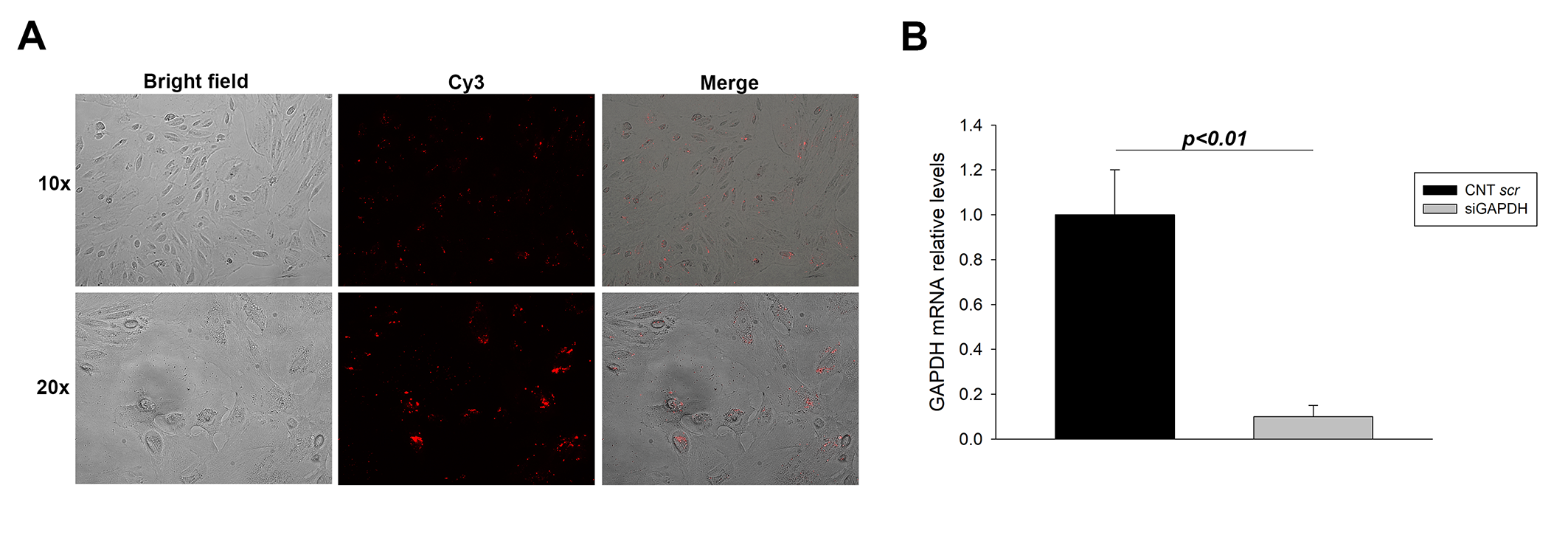

Supplement: Supplementary file 1 [file biology-12-00690-s001.zip › Supplementary Figure S1.tif]

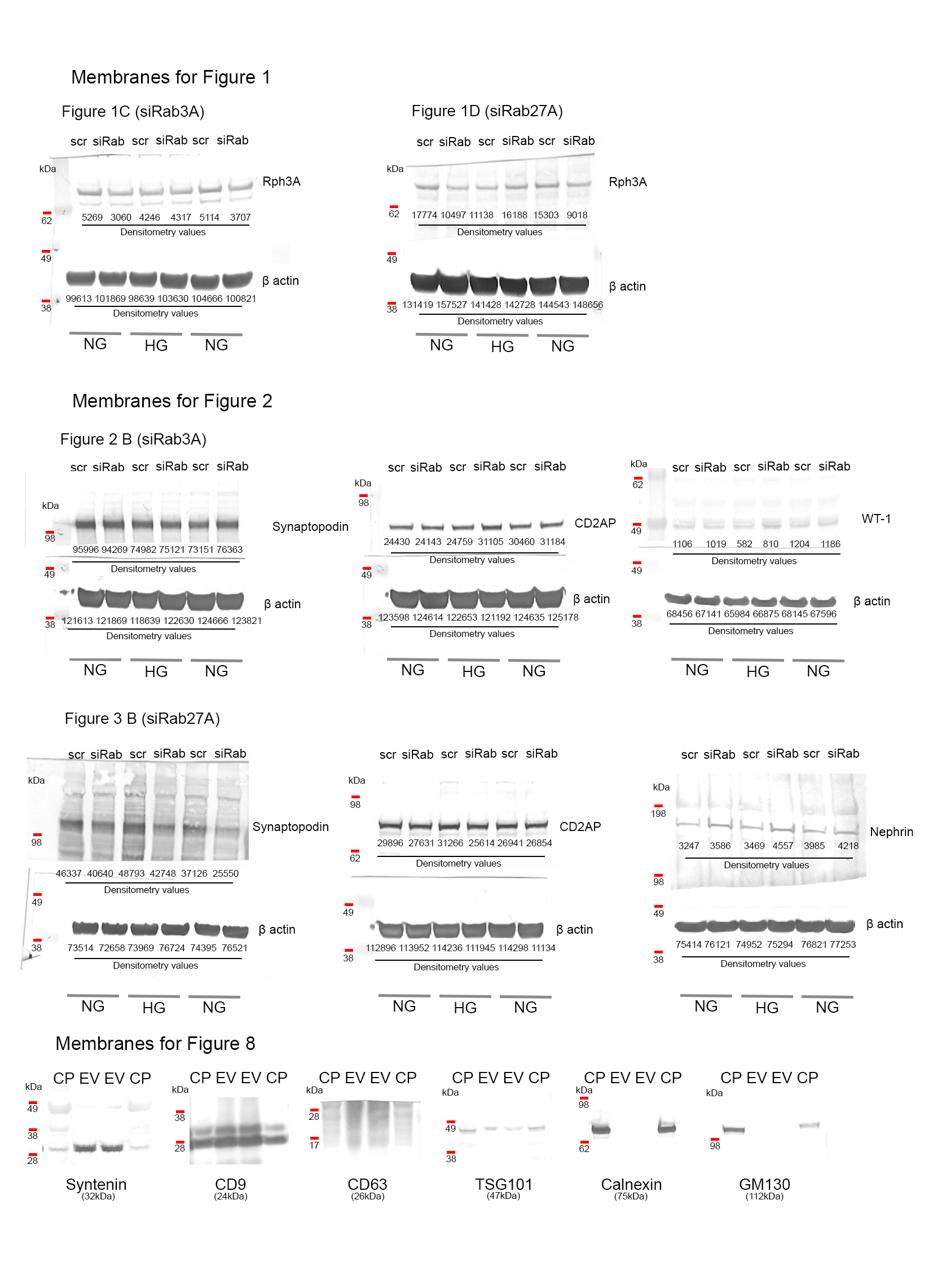

Supplement: Supplementary file 1 [file biology-12-00690-s001.zip › Supplementary Figure S2.tif]
